# Supplementary material for: Causal Relationship between Aging and Anorexia Nervosa: A White-Matter-Microstructure-Mediated Mendelian Randomization Analysis
Source: Biomedicines. 2024 Aug 16;12(8):1874. doi: 10.3390/biomedicines12081874 (PMC11351342; doi:10.3390/biomedicines12081874)
Supplement: Supplementary file 1 [file biomedicines-12-01874-s001.zip › Supplementary Figure/Figure S8/Leaveoneout plot of MR analysis for TL to ubm-b-2012.pdf]

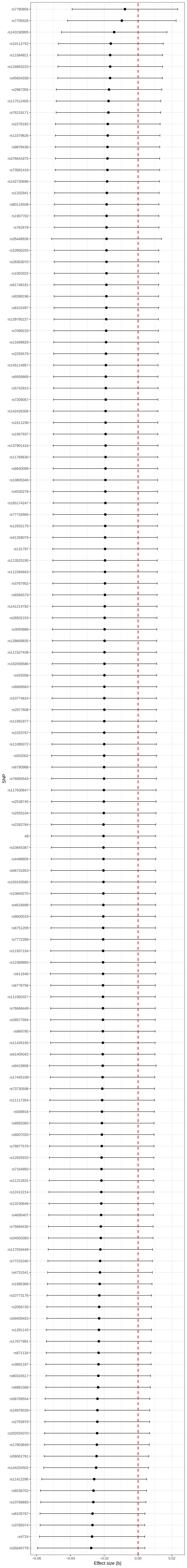

rs7790856
rs7705526
rs143190905
rs10112752
rs11584821
rs116863223
rs45604339
rs2967355
rs117512405
rs78219171
rs2276182
rs11579626
rs9878436
rs376641875
rs73581419
rs142730696
rs1332941
rs80116508
rs1907702
rs762679
rs35446936
rs10905255
rs28363070
rs1003322
rs61748181
rs9398196
rs8102497
rs139795227
rs7099229
rs11699829
rs2293579
rs145114957
rs6659669
rs5742915
rs7209057
rs142426306
rs1611236
rs1957937
rs137901416
rs11769630
rs9940099
rs10805346
rs4530278
rs185174247
rs77732866
rs12932178
rs41269079
rs131797
rs113525195
rs112394943
rs3767952
rs6584579
rs141214782
rs28502153
rs3093888
rs139669835
rs111527438
rs182059586
rs429358
rs6669563
rs10774624
rs2977608
rs11991877
rs1023767
rs11085072
rs932002
rs6790986
rs76065543
rs117630647
rs2538745
rs2555104
rs2282764
All
rs10845387
rs4498805
rs66731853
rs150150565
rs10840270
rs4616688
rs9600019
rs6751209
rs7772289
rs11557154
rs12369950
rs611646
rs6776756
rs111950327
rs76666449
rs28577594
rs869785
rs11426156
rs61405042
rs9419958
rs17445108
rs73730598
rs11117354
rs939916
rs9955360
rs6007020
rs79977579
rs12925933
rs7164950
rs11212631
rs12412214
rs13230646
rs4695407
rs75664430
rs34550383
rs117034449
rs77231040
rs4731541
rs1985369
rs10773176
rs2056726
rs59409453
rs1291143
rs17677991
rs871134
rs3891167
rs80324517
rs6881568
rs56799554
rs16978028
rs2763979
rs202034370
rs17803849
rs56061761
rs144204502
rs11412296
rs6536702
rs10768683
rs8105767
rs3785074
rs4724
rs35640778
SNP
-0.06
-0.04
-0.02
0.00
0.02
Effect size (b)
